# Reconstructive dosimetry for cutaneous radiation syndrome

**DOI:** 10.1590/1414-431X20144337

**Published:** 2015-05-08

**Authors:** C.M.A. Lima, A.R. Lima, Ä.L. Degenhardt, N.J. Valverde, F.C.A. Da Silva

**Affiliations:** 1 Instituto de Radioproteção e Dosimetria, IRD/CNEN, Rio de Janeiro, RJ, Brasil; 2 Fundação Eletronuclear de Assistência Médica, Rio de Janeiro, RJ, Brasil

**Keywords:** Radiological accident, Radiation injury, Cutaneous radiation syndrome, Reconstructive dosimetry, Industrial radiography, Monte Carlo method

## Abstract

According to the International Atomic Energy Agency (IAEA), a relatively significant number of radiological accidents have occurred in recent years mainly because of the practices referred to as potentially high-risk activities, such as radiotherapy, large irradiators and industrial radiography, especially in gammagraphy assays. In some instances, severe injuries have occurred in exposed persons due to high radiation doses. In industrial radiography, 80 cases involving a total of 120 radiation workers, 110 members of the public including 12 deaths have been recorded up to 2014. Radiological accidents in industrial practices in Brazil have mainly resulted in development of cutaneous radiation syndrome (CRS) in hands and fingers. Brazilian data include 5 serious cases related to industrial gammagraphy, affecting 7 radiation workers and 19 members of the public; however, none of them were fatal. Some methods of reconstructive dosimetry have been used to estimate the radiation dose to assist in prescribing medical treatment. The type and development of cutaneous manifestations in the exposed areas of a person is the first achievable gross dose estimation. This review article presents the state-of-the-art reconstructive dosimetry methods enabling estimation of local radiation doses and provides guidelines for medical handling of the exposed individuals. The review also presents the Chilean and Brazilian radiological accident cases to highlight the importance of reconstructive dosimetry.

## Introduction

The relationship between industrial radiography and radiological accidents is now well recognized, which makes this industrial practice one of the highest potential risks for human health. According to the International Atomic Energy Agency (IAEA) and the United Nations Scientific Committee on the Effects of Atomic Radiation (UNSCEAR) (1,2), so far there have been 80 different radiological accidents involving 120 radiation workers, 110 members of the public and 12 deaths. Brazilian data includes 5 serious radiological accidents that affected 7 radiation workers and 19 members of the public, who developed cutaneous radiation syndrome (CRS - also called as "local radiation injury" or "radiation burn") in hands and fingers (3,4). The Brazilian gammagraphy accident that occurred in May 2000 involved an operator performing routine exposures with a ^60^Co apparatus containing a 2.11 TBq source. He received a localized exposure when his left hand was very close to the radioactive source for approximately 30 s (5,6).

Estimation of accidental radiation doses by clinical parameters is generally less accurate. Therefore, several techniques based on physical, computational and biological dosimetry methods have been used in the last decade for reconstructive dosimetry to evaluate such accidental radiation doses.

This article reviews the state-of-the-art reconstructive dosimetry for estimating localized radiation doses, and also focuses on the dosimetry results of the Brazilian gammagraphy accident. The effective doses in the Brazilian gammagraphy accident were estimated using physical dosimetry with individual film badge monitor processing and biological dosimetry based on chromosomal aberrations. Physical dosimetry with thermoluminescent dosimeters on a hand phantom, and computational dosimetry with the Brazilian software "Visual Monte Carlo Dose Calculation" (http://www.vmcsoftware.com/) were used to estimate the equivalent doses for localized injuries observed on the clinical manifestation in terms of cutaneous radiation. The efficacy of the estimation methods have also been compared.

## Description of different reconstructive dosimetry methods

### Physical dosimetry

Physical methods for retrospective dosimetry conventionally include the electron paramagnetic resonance (EPR), thermoluminescence (TLD), optically stimulated luminescence (OSLD) and nuclear activation techniques. These methods are typically used in physical science studies. However, the physical methods do not show any biological response even when performed in biological tissues, such as hair, fingernails and tooth enamel bone (7).

### Electron paramagnetic resonance

The EPR technique (7) provides an estimate of absorbed radiation dose by detecting paramagnetic centers, such as radicals or point defects that are specifically generated by ionizing radiation. EPR spectroscopy is the most advanced physical method for retrospective dose assessment in tooth enamel of individuals (8,9). It has been extensively used for historical and chronic exposures (10), such as in the case of atomic bombs in Hiroshima, (11), Chernobyl (12) and the Southern Urals radiation incidents (13). In acute exposure and severe accidents, if bone biopsies are available, bone samples can be used especially for localized or heterogeneous irradiation cases (14). However, tooth enamel and bones require invasive collection. Hence, other materials, such as sugar, plastics, glass, wool, cotton, hair and nails that can be collected through non-invasive procedures are more suitable for fortuitous EPR dosimetry.

Preparation of samples for EPR dosimetry is relatively simple. Depending on the material, a single measurement can take from several minutes to a few hours. EPR is advantageous as the readout is non-destructive allowing for repeated measurements of the same sample. However, EPR spectrometers are expensive and require highly skilled personnel for their operation. EPR detection limits vary widely between ∼100 mGy for tooth enamel and 10 Gy for cotton. Data interpretation can suffer from the presence of background non-radiation-induced EPR signals. There are a few studies on the effect of different qualities of radiation on some of the above-mentioned materials (7). EPR dosimetry is particularly suitable for application after local or non-uniform exposures as the dose heterogeneity can be assessed by using several materials from different parts of the body.

### Luminescence dosimetry

Ionizing radiation absorbed by an insulator or a semiconductor produces free charge carriers that can be trapped at lattice defects of the material. Luminescence dosimetry (7) is based on the stimulated emission of light from these materials by release of the trapped charge carriers and subsequent recombination. Stimulation is performed either thermally (TLD) or optically (OSLD).

Quartz extracted from bricks and other fired-building materials is currently the main mineral used for retrospective luminescence dosimetry purposes. Sample preparation techniques and measurement protocols of quartz dosimeter are well established, which may take more than one day. Various studies have been performed with quartz to evaluate the external exposure in the area of Chernobyl, in areas affected by fallout from the Semipalatinsk and Nevada nuclear test sites and in the Southern Urals (15). The minimum detectable doses that can be obtained from bricks a few decades old is in the order of 20-25 mGy.

The possibility of using quartz extracted from unfired building materials (mortar, concrete, etc.) was also tested (15). However, in such cases, a detection limit higher than 100 mGy was observed. Recently, in addition to quartz, other phosphors, found either in the urban environment or in materials carried on or close to the body by the general population (16), have also been studied for dosimetry application. Examples of such materials include memory chip modules from telephones, ID, health insurance, cash and credit cards (15-18), ceramic resistors of portable electronic devices such as mobile phones (18,19), materials used for dental restoration (15,20), tooth enamel (21,22), household and workplace chemicals (23,24) and glass (25). Inorganic dust extracted from natural materials or personal items has also been investigated. Most of these items show a linear dose-response over a wide dose range. The radiation sensitivity and time stability of the response strongly depend on the type of material, but detection limits of the order of 10 mGy can be achieved for most materials. However, for tooth enamel, the detection limits are presently more in the range of 1-5 Gy.

### Activation techniques

Neutron activation techniques (7) are based on the measurement of radioactivity induced by neutron interaction with biological tissues, such as blood, hairs and nails or metallic elements, such as coins, jewelry or belt buckles, used by the victims. Activation techniques can be used in emergency management of critical accidents and in dose reconstruction, many years following exposure to neutrons, such as for atomic bomb survivors.

### Computational dosimetry

The computational dosimetry methods are generally based on analytical and numerical calculations. The Monte Carlo method, such as dosimetry by numerical computer code MCNPX and the computational program based on voxel anthropomorphic phantom in combination with Monte Carlo simulation are currently the most widely used methods.

### Analytical dose reconstruction (‘time and motion’ calculations)

The techniques applied for analytical reconstruction of individual doses following radiation accidents have been well established for decades (7). A state-of-the-art analytical method, known as realistic analytical dose reconstruction with uncertainty estimation (RADRUE), was developed by an international group of experts (26) for estimation of external exposures of Chernobyl clean-up workers. The method is based on a time-and-motion approach so that the subject's exposure can be estimated as time spent in certain locations multiplied by exposure rate at those locations, taking into account the applicable shielding factors. Stochastic modeling is applied to dose calculations in order to estimate uncertainties. It can be easily expanded to any other accidental situation, where exposure rates are mapped and individual exposure itineraries are available. The RADRUE program does not include a dose threshold, and is applicable to a large range of exposures. It is suitable for air kerma and organ dose reconstruction using embedded exposure-to-dose conversion coefficients (e.g., red bone marrow, thyroid). However, neither partial-body exposures nor internal exposures are covered by RADRUE. The method has been applied for case-control studies of hematological malignancies and thyroid cancer (27).

### Dose reconstruction by numerical approaches

A large variety of numerical tools are used to estimate dose retrospectively in individuals (7). Most of these tools are based on Monte Carlo radiation transport codes to simulate the transport of particles in a defined geometry, and thus a dose map can be constructed. It has been used for a wide range of applications. It is possible to estimate dose distributions in the organism, effective doses or doses to specific organs with the help of numerical phantoms of human body for planned or accident situations, for radiation protection purposes or dose reconstruction for overexposed individuals. These approaches have recently been used for accidents during interventional radiology procedures, industrial irradiation processing and events with lost or orphan sources (28,29). In cases of localized and severe irradiations, dose distribution calculations enable the surgical removal of lethally exposed tissue before radiation necrosis occurs ("dosimetry guided surgery"). In such a case, calculations are performed with voxel phantoms derived from MRI or CT scans to take into account the individual anatomy of the patient.

### Monte Carlo method

Many computational tools use Monte Carlo code to estimate the absorbed dose in the organism for evaluating the biological consequences of an overexposure. SESAME is one such tool from France, which is dedicated to dose reconstruction of radiological accidents based on anthropomorphic voxel phantoms built from real medical images of the victim in association with the MCNP Monte Carlo code. It is a very powerful tool since it offers the possibility to simulate realistically the victim and the environment for dose calculations in various accidental situations (30). Another tool is the Brazilian software named "Visual Monte Carlo Dose Calculation", which was also developed using the Monte Carlo method and a human body voxel simulator. The Visual Monte Carlo (VMC) transports photons, protons and alpha particles through inhomogeneous geometries, mostly through voxel geometries. The VMC software enables the calculation of absorbed dose received by each organ and tissue for determining effective dose, according to the International Commission on Radiological Protection (ICRP) guidelines (31). The VMC code has been effective in quick estimation of the doses of radioactive sources in planned or accidental exposures situations, especially for cases of handling radioactive sources. The code can be used with the source near the surface of any part of the body with accurate dose estimation (5,6,32).

### Cytogenetic techniques

The most commonly used biological dosimetry method is the cytogenetic technique, used mainly for whole-body dose estimation (7). It is based on the analysis of chromosomal aberrations in peripheral blood lymphocytes (PBL) induced by ionizing radiation. The applicability of the available assays is based on whether the chromosomal damage is stable or not. Dicentric, premature chromosome condensation fragment and micronucleus frequencies fall with the turnover of lymphocytes, enabling application of these assays for dose assessment in more recent exposures. For exposures that have taken place years or decades ago or are chronic in nature, the choice is fluorescence *in situ* hybridization (FISH) to detect stable translocations. Dicentric chromosomes are almost exclusively induced by ionizing radiation.

Dicentric frequencies in PBL show a clear linear quadratic dose-effect relationship up to 5 Gy for acute photon exposures. Numerous studies on both low and high linear energy transfer (LET) radiations have demonstrated that exposures *in vitro* and *in vivo* produce similar yields of dicentrics per unit dose. The spontaneous frequency of dicentrics is very low in the healthy general population (about one dicentric per 1000 cells). Due to this low background, the sensitivity of the dicentric assay is relatively good and able to detect whole-body doses down to about 0.1 Gy from the analysis of 500-1000 metaphase spreads (33). Ideally, the dicentric assay is performed on blood samples within a few days of the exposure. Blood sampling after weeks or months requires the intrinsic exponential removal rate of dicentrics (halftime between 6 months and 3 years) to be taken into account. Mathematical procedures exist to modify the dose-squared coefficient in case of dose protraction or to provide dose estimation after partial-body exposure (33).

## Acute and cutaneous radiation syndromes

Acute radiation syndrome (ARS) may occur when the whole-body or a significant part of it (at least about one-third) receives a high penetrating radiation exposure. ARS may manifest as 3 types: i) hematopoietic, ii) gastrointestinal, and iii) cerebrovascular ARS. Their specific dose thresholds for irradiation in a maximum two-day period is >1 Gy for hematopoietic, 6-10 Gy for gastrointestinal, and >20 Gy for cerebrovascular ARS.

Very briefly, the hematopoietic type of the ARS is characterized by damage to the proliferating stem cells of the bone marrow with consequent depletion of circulating mature blood cells. The pathophysiological consequences include, in a dose-dependent degree of severity, increased susceptibility to infection, bleeding, anemia, and decreased immunity. In the gastrointestinal type, irradiation inhibits the renewal of cells lining the digestive tract. The consequences vary depending on the exposed region and extent of damage. The depletion of the epithelial lining may lead to severe denudation of the mucosa, massive loss of fluid and electrolytes, septicemia, hypovolemic shock and death. The cerebrovascular type of ARS is due to microvascular damage to the central nervous system (CNS) with untreatable vasoplegia, irreversible shock, and death.

The LD_50/60_ (50% of the affected individuals surviving at 60 days after radiation exposure) for ARS patients under excellent medical assistance (multidisciplinary and intensive care, admission to facilities with laminar flow and high efficiency particulate air (HEPA) filtering, availability of last generation antibiotics and growth factors, etc.) is in the range of 5 to 6 Gy. Cerebrovascular ARS is always fatal within a few days after exposure (34,35). It is not under the primary scope of this paper.

CRS (34,35) is a set of manifestations caused by pathological changes in the skin and underlying structures. It is caused by the absorption of radiation doses above certain thresholds, from a radiation source outside the body (see Table 1). CRS has a spectrum of manifestations and its severity depends on a number of conditions. They include the absorbed dose by the skin and factors such as doses-rates, geometry of exposure, affected area of the body, and energy of the radiation. As the skin consists of the epidermis, dermis, hypodermis and other structures, such as vessels and nerve endings, with different radio-sensitivities, the consequent injury may be more superficial on the skin, but can also be expressed in deeper tissues. The earliest response of the skin to irradiation is a transient primary erythema, which may appear within hours after exposure, resulting from capillary dilatation caused by the release of histamine and other vasoactive peptides. In cases of high or very high-localized doses, skin manifestations, such as erythema, edema and blistering may appear after a short time (few days). This serves as a clinical indication of poor prognosis, and therefore, an accurate dosimetry should be promptly established to guide proper medical intervention. Usually, after the primary erythema, there exists a variable latent phase without evident medical manifestation depending on the dose (higher the dose, shorter will be the latent period). The latent period is followed by the manifestation of clinical signs and symptoms in accordance with the threshold dose ranges and energy of the radiation (Table 1). In cases of high-doses, CRS manifestations, recurrences and sequelae may appear even months or years after exposure in spite of apparent healing. Skin cancer is also a possibility, many years after exposure.

**Table:**
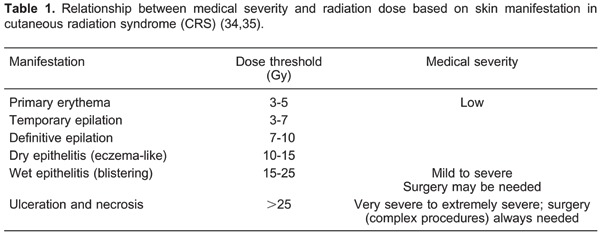

## The Chilean radiological accident

On 14 December 2005, a serious radiological accident occurred at a cellulose plant under construction in Chile when a radioactive source containing ^192^Ir (3.33 TBq activity) fell out of a gammagraphy equipment unnoticed (36). One of the exposed workers developed a serious CRS on his left buttock. The patient exhibited erythema of about 4 cm deep within 5 h of exposure. Two days later, erythema, blister, edema and an eschar were evident on the affected area. These manifestations were clearly indicative of an extremely high local radiation dose. The calculations done in France estimated a maximum local dose of 2,000 Gy in the center of the lesion and a very sharp gradient of the dose as a function of both depth and surface distance (36). Based on a dosimetric map, the patient was operated in the Percy Military Hospital, Clamart, France. The first surgery consisted of an excision measuring 5 cm in depth by 10 cm in diameter on the buttock. Also, mesenchymal stem cells (MSC) were injected in the affected areas. MSCs can be mainly obtained from the bone marrow and the adipose tissue. They have a high proliferating ability, and are multipotential, especially for structural tissues (as they can differentiate into bone, cartilage, muscle, stroma, tendon and adipocytes). They secrete cytokines and soluble factors and induce immunotolerance. The patient's recovery was excellent. Thereafter, dosimetry guided surgery with simultaneous injection of MSCs has been used for the treatment of about 10 other individuals with severe CRS with positive results (N.J. Valverde, personal communication). The Chilean case is an excellent example of how reconstructive dosimetry can be important for medical procedures related to radiation cases.

## The Brazilian radiological accident

In May 2000, an operator at an industrial radiography company in Brazil suffered hand injuries from exposure to a ^60^Co radioactive source with 2.11 TBq activity. Reconstructive dosimetry was conducted by Da Silva (5,6), together with the accident investigation. The effective and absorbed dose estimations were performed using all three different techniques and approaches (physical dosimetry, computational dosimetry and biological dosimetry). The physical method was based on film badge individual monitoring and irradiation of a simulator of a left hand containing thermoluminescent dosimeters. The biological method used the cytogenetic analysis and the computational method used the Brazilian Monte Carlo calculation code "Visual Monte Carlo Dose Calculation -VMC" with human body voxel simulator. Clinical observation of the sequential development of lesions, especially in fingers and left hand, were useful for initial dose estimation.

The physical dosimetry method was performed to estimate the effective dose (whole-body) and the absorbed dose in the hand (local). The effective dose was estimated by the operator's film badge processing provided by the approved individual monitoring laboratory (IRD/CNEN laboratory). The absorbed doses for the operator's hand were calculated by using a left hand simulator with thermoluminescent dosimeters. This simulator was composed of a latex glove, internally filled with solid flakes of expanded polystyrene, which made it adjustable to the suitable format of the left hand. Expanded polystyrene is not tissue equivalent; so it did not affect the experiment significantly. The external surface of the glove was attached with fifteen LiF-100 thermoluminescent dosimeters of dimensions 3.2×3.2×0.89 mm in order to map the absorbed doses received by the operator's left hand. The irradiations were simulated for a 2.11 TBq ^60^Co source for 30 s (5,6).

For cytogenetic analysis, the operator's blood sample was collected 15 days after the accident and 1000 cells were scored at the IRD/CNEN Cytogenetic Laboratory.

For the computational method, the hand simulator configuration in Brazilian VMC code was based on information from a whole-body magnetic resonance image scan of a real man (NORMAN voxel simulator), adjusted to make the simulator to the same height (1.76 m) and mass (73 kg) as the reference man. The size of each voxel was 2.08×2.08×2.02 mm with its tissue type (e.g., bone, muscle). Voxels were defined in the hand voxel simulator at the positions corresponding to the respective TLDs. The TLDs were represented by a matrix composed of 25 voxels in the format of 5×5×1 voxels. The irradiations were simulated for a 2.11 TBq ^60^Co source, for 30 s. The results were obtained after 8 h of simulation, when 15 million photon histories were run in order to obtain the superficial absorbed doses in each phalanx of the left hand voxel simulator.

The clinical dose indicators were made based on observation of lesion characteristics including the evolution to radiation-induced ulcerations. Taking into account the development of skin manifestations, such as edema and erythema on the 8th day post-exposure, blisters mainly on the left thumb and index fingers on the 21st day, and dry desquamation on both hands on the 23rd day, an initial gross estimation of the absorbed doses for the operator's hand was possible.

The results of the reconstructive dosimetry of the Brazilian industrial radiological accident (5,6) are reproduced in Tables 2and3.

**Table:**
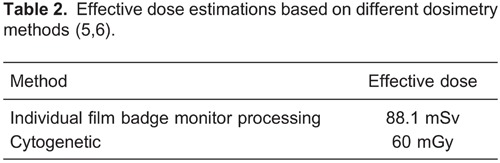

**Table:**
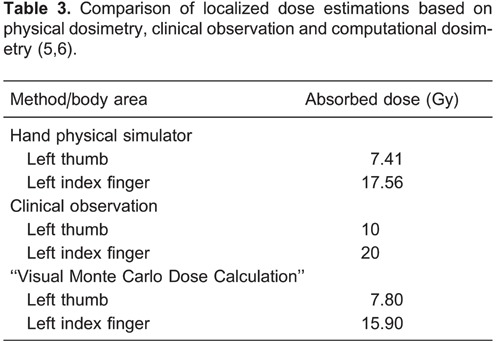

The absorbed doses on the operator's left hand estimated through physical dosimetry methods and the VMC program were comparable fairly well with the clinical observation based on the localized radiation effects presented on the operator's hand.

## Conclusions

This review focused on discussing the state-of-the-art physical, biological and computational reconstructive dosimetry methods commonly used in the last 10 years to manage radiation exposure cases. The most important physical reconstructive dosimetry methods are the luminescence methods including thermoluminescence and optical stimulated luminescence, and electron paramagnetic resonance. The computational methods are based on Monte Carlo simulation, such as the numerical code MCNPX and a program based on Voxel anthropomorphic phantom. The most common biological method is the cytogenetic technique based on the analysis of chromosome aberrations, especially dicentrics (this method is more useful for whole-body exposures). Clinical parameters for dose estimations are based on observation of the development of signs and symptoms, but they are much more reliable for ARS (as the time for the onset and the severity of prodromal manifestations like nausea and vomiting) than for CRS.

The VMC software can be considered suitable for estimating the distribution of doses to hands in radiological accidents. Initial dose estimates through the observation of clinical parameters can be used just as a preliminary reference, especially in severe cases of CRS contemplating the use of complex surgical procedures.

## Recommendations

Although several methods are now available for estimating doses of accidental ionizing radiation exposures of individuals, it is recommended to use the most accurate dosimetric evaluation method. This is especially important in cases where CRS develops or is likely to develop because the adequate surgical procedure that follows must be made timely before the development of necrosis. The isodoses curves (doses to the deeper structures) will provide *a priori* information to the surgeon about the tissues that are prone to exhibit necrosis.

The medical management of serious cases of CRS demands a multidisciplinary approach involving physicians, physicists, biologists, health personnel, etc. It is strongly recommended to alert these professionals about the importance of integrated work in such situations.
